# Suprascapular Nerve Entrapment Caused by Protrusion of an Intraosseous Ganglion of the Glenoid into the Spinoglenoid Notch: A Rare Cause of Posterior Shoulder Pain

**DOI:** 10.1155/2017/1704697

**Published:** 2017-05-23

**Authors:** Daichi Ishimaru, Akihito Nagano, Nobuo Terabayashi, Yutaka Nishimoto, Haruhiko Akiyama

**Affiliations:** Department of Orthopaedic Surgery, Gifu University School of Medicine, Yanagido 1-1, Gifu, Gifu Prefecture, Japan

## Abstract

We describe a case of suprascapular nerve entrapment caused by protrusion of an intraosseous ganglion of the glenoid into the spinoglenoid notch. A 47-year-old man with left shoulder pain developed an intraosseous cyst in the left glenoid, which came into contact with the suprascapular nerve. The area at which the patient experienced spontaneous shoulder pain was innervated by the suprascapular nerve, and 1% xylocaine injection into the spinoglenoid notch under ultrasonographic guidance relieved the pain. Therefore, we concluded that the protrusion of an intraosseous cyst of the glenoid into the spinoglenoid notch was a cause of the pain, and performed curettage. Consequently, the shoulder pain was resolved promptly without suprascapular nerve complications, and the cyst was histologically diagnosed as an intraosseous ganglion. This case demonstrated that the intraosseous ganglion of the glenoid was a benign lesion but could be a cause of suprascapular nerve entrapment syndrome. Curettage is a useful treatment option for a ganglion inside bone and very close to the suprascapular nerve.

## 1. Introduction

The suprascapular nerve is a mixed motor and sensory nerve originating from the brachial plexus. It passes between the suprascapular notch and superior transverse scapular ligament into the posterior surface of the scapula, which dominates the supra- and infraspinatus muscles. Suprascapular nerve entrapment is an uncommon but significant cause of shoulder pain [1], and a ganglion originating from the soft tissues around the spinoglenoid notch has been reported to be a cause of suprascapular nerve entrapment [2]. However, suprascapular nerve entrapment caused by an intraosseous ganglion occurring in the glenoid is rare. Here, we present a case of suprascapular nerve entrapment caused by an intraosseous ganglion in the glenoid, which was treated with curettage of the ganglion.

## 2. Case Presentation

**Figure 1:**
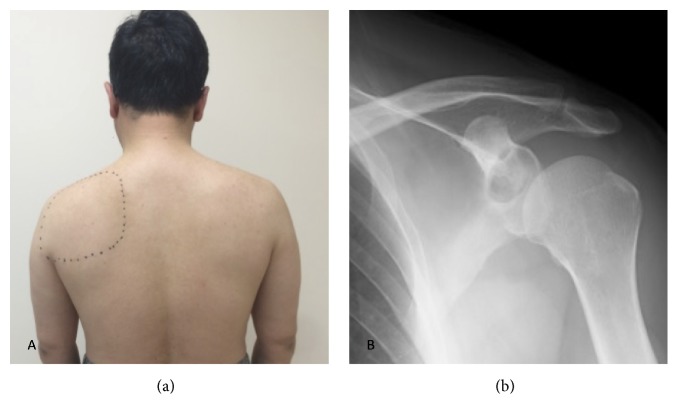
(a) Clinical photograph before the surgery shows a dotted circle at the left shoulder that indicates the area at which the patient complained of pain. The area includes the supra- and infraspinatus muscles and the suprascapular nerve. (b) Radiograph of the left shoulder shows a radiolucent cystic lesion in the superior glenoid.

**Figure 2:**
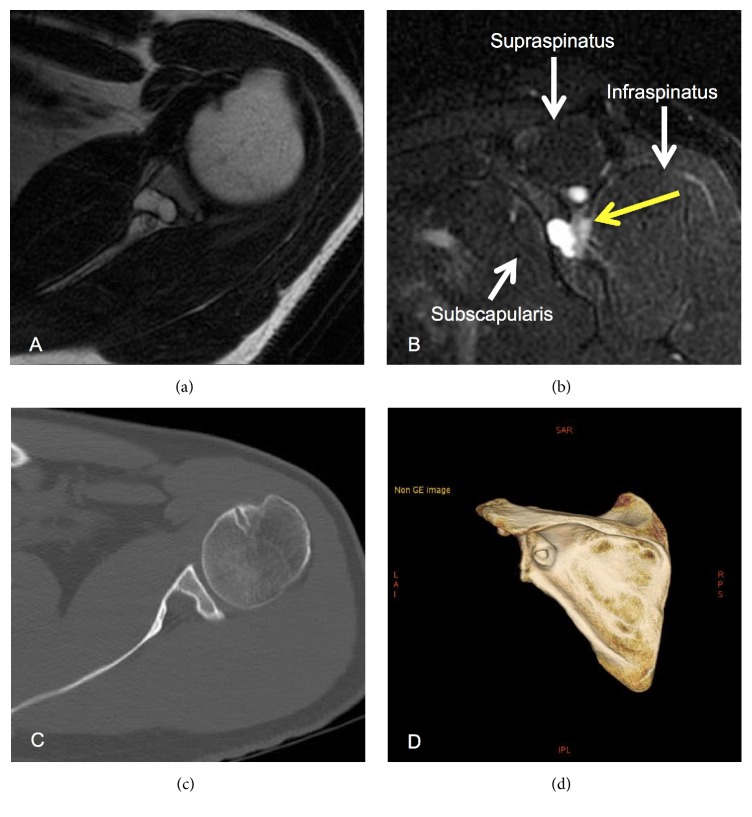
(a) Magnetic resonance (MR) T2-weighted axial image of the left shoulder shows a high intensity area at the glenoid. (b) MR T2-weighted sagittal image of the left shoulder shows that the intraosseous lesion is linked to the spinoglenoid notch. (c, d) Computed tomography (CT) axial and 3-dimensional CT images of the left shoulder show a bone cystic lesion of the glenoid with cortical bone destruction linked to the spinoglenoid notch.

A 47-year-old man with left shoulder pain lasting for more than 2 years was referred to our hospital because a radiolucent cystic lesion of the glenoid was observed on plane radiography of the left shoulder. He had no history of dislocation or trauma of the shoulder joint. On visiting our hospital, he complained of continuous posterior shoulder pain at rest; however, he could move his shoulder actively at 160 degrees of flexion and abduction, 60 degrees of external rotation at the side, and 90 degrees of external rotation at abduction and could maintain muscle strength around the shoulder on manual muscle testing. He did not show sensory and motor paralysis of the supra- and infraspinatus muscles and did not exhibit muscle atrophy on visual examination. The area at which he experienced spontaneous shoulder pain was innervated by the suprascapular nerve (Figure 1(a)). Plain anteroposterior radiography of the left shoulder showed a circular lesion with marginal osteosclerosis at the glenoid (Figure 1(b)). Magnetic resonance imaging (MRI) showed an osteolytic cystic lesion with very high intensity on T2-weighted images and low intensity on T1-weighted images, and the lesion was located at the posterior cranial portion of the glenoid and partially extended to the spinoglenoid notch (Figures 2(a) and 2(b)), and there were no findings about fatty change and intramuscular edema in supra- and infraspinatus muscles. Computed tomography (CT) showed a circular cystic lesion with marginal osteosclerosis and cortical bone destruction of the posterior glenoid at the spinoglenoid notch (Figures 2(c) and 2(d)). Based on the findings of MRI and CT, an intraosseous ganglion, cyst of degenerative disease, giant cell tumor, aneurysmal bone cyst, and chondroblastoma of the glenoid were suspected. Needle aspiration for the cyst was performed using an 18-gauge needle under ultrasonographic guidance; however, no aspirate was obtained. After injecting 1% xylocaine into the spinoglenoid notch under ultrasonographic guidance, the posterior shoulder pain resolved. Based on these findings, it was considered that the intraosseous cyst in the glenoid compressed the suprascapular nerve at the spinoglenoid notch and induced the posterior shoulder pain, though a nerve conduction study for suprascapular nerve was not performed. Therefore, curettage of the cyst was performed.

**Figure 3:**
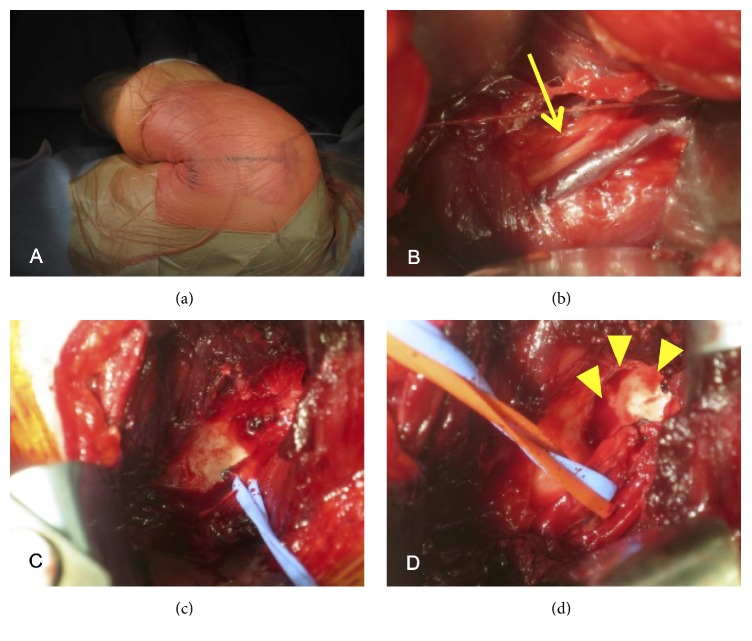
(a) Surgery for the intraosseous ganglion is performed in the right lateral position. A 10 cm skin incision is made at the posterior glenohumeral joint. (b) Intraoperative photograph of the posterior shoulder shows the suprascapular nerve after splitting the infraspinatus (yellow arrow). (c) Intraoperative photograph shows a mucinous cyst wall after shifting the suprascapular nerve laterally. (d) Intraoperative photograph shows the bone cavity of the glenoid after curettage of the cyst (yellow arrowheads).

Curettage was performed under general anesthesia in the right lateral position (Figure 3(a)). A skin incision of approximately 10 cm was made along the glenoid on the lateral side of the scapula. The spinoglenoid notch was directly visualized after splitting the teres minor and infraspinatus muscles, and the suprascapular nerve and cyst were identified at the spinoglenoid notch (Figure 3(b)). The suprascapular nerve was raised upward by the cyst wall present below the nerve. The nerve was stretched and edematous, and it highly adhered to the cyst wall. After releasing the nerve gently from the cyst (Figure 3(c)), an incision was made at the cyst wall, and gelatinous material flowed out from the cyst. Thus, the intraosseous cyst was considered to be an intraosseous ganglion. The suprascapular nerve was retracted gently, and curettage was performed until the bone surrounding the cyst was completely exposed (Figure 3(d)).

**Figure 4:**
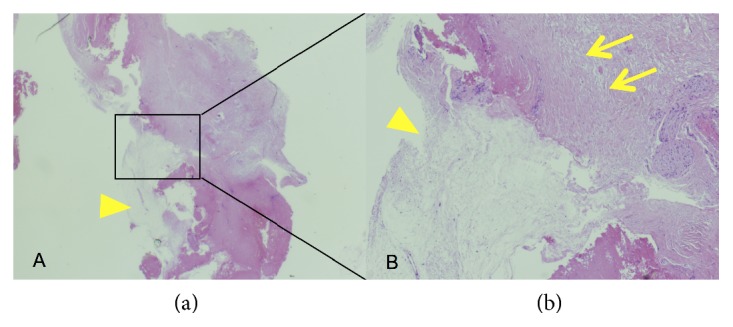
(a, b) Microscopic section of the cyst wall shows that the wall contained connective tissue, including collagen fibers and a few fibroblasts, and that the inner layer of connective tissue had myxoid change. The yellow arrow indicates the myxoid area, and the yellow arrowheads show connective tissue, including collagen fibers and a few fibroblasts (hematoxylin and eosin staining; A: ×12.5 magnification, B: ×50 magnification).

Postoperatively, the patient's shoulder pain resolved promptly. Histological examination revealed that the cyst wall contained connective tissue, including collagen fibers and a few fibroblasts, and that the inner layer of connective tissue exhibited myxoid change (Figures 4(a) and 4(b)). These findings were consistent with an intraosseous ganglion.

At the 1-year follow-up, the patient was asymptomatic and had no functional deficits and osteoarthritic changes of the glenohumeral joint were not observed on CT, but recurrence of the ganglion was observed at the glenoid on MRI.

## 3. Discussion

We experienced a rare case of suprascapular nerve entrapment caused by protrusion of an intraosseous ganglion of the glenoid into the spinoglenoid notch. The patient's posterior shoulder pain resolved after successful curettage of the ganglion.

An intraosseous ganglion is a benign bone lesion but is considered a neoplasm, which is similar to a ganglion occurring in soft tissue [3, 4]. Few reports have presented the characteristics of an intraosseous ganglion, such as its incidence and etiology, and this ganglion is considered to be relatively rare. It mainly occurs in middle-aged people, and the most common site is the lower end of the tibia [4, 5]. To our knowledge, 19 cases of intraosseous ganglion of the glenoid have been reported in the English literature [4–10]. Among these cases, 2 cases showed a fracture around the ganglion [7, 8] and only 1 case was accompanied with suprascapular nerve entrapment, which is similar to our case [9].

Recently, a simple and safe less invasive arthroscopic approach was reported for patients with spinoglenoid ganglion cysts [11]. Additionally, the previously reported case of an intraosseous ganglion accompanied with suprascapular nerve entrapment was treated with needle aspiration under arthroscopy [9], and posterior shoulder pain and muscle strength weakness of the shoulder resolved. In our case, we used curettage and did not consider arthroscopic treatment for various reasons. First, based on the radiographic finding of bone cortex destruction at the spinoglenoid notch, the differential diagnosis included an intraosseous ganglion, giant cell tumor, aneurysmal bone cyst, and chondroblastoma [12, 13], so we needed a specimen to perform histological examination. Second, needle aspiration or incision under arthroscopy was speculated to be arduous because the main part of the ganglion was located inside the glenoid and it was apart from the glenohumeral joint cavity. Third, the suprascapular nerve was found to be very close to the cyst at the spinoglenoid notch on MRI. Consequently, the intraosseous cyst was identified as a ganglion based on the finding of mucoid viscous effluent on curettage during surgery and the pathological findings. Furthermore, in surgery, we could identify the intraosseous cyst and suprascapular nerve and protect the nerve under direct vision; nevertheless, the intraosseous cyst compressed the nerve and adhered to it causing edema. As a result, we could perform curettage of the cyst wall, which relieved the patient of the symptom. However, the ganglion relapsed at the 1-year follow-up on MRI, suggesting that curettage for the ganglion was inadequate and en bloc resection would be necessary if the symptoms of the left shoulder recur.

Generally, the bone cortex and periosteum act as substantial physical barriers, and a ganglion occurring in soft tissue has difficulty penetrating into the bone. Therefore, in the present case, it was believed that the ganglion did not occur in the soft tissue around the spinoglenoid notch and then penetrate into the glenoid, but it occurred primarily inside the glenoid and protruded into the spinoglenoid notch accompanied with bone destruction over a long period.

We reported a case of suprascapular nerve entrapment caused by protrusion of an intraosseous ganglion of the glenoid into the spinoglenoid notch, which is a rare cause of posterior shoulder pain. We performed curettage with protection of the suprascapular nerve, and the patient's pain resolved completely without suprascapular nerve complications. We believe that open surgery including curettage is a useful treatment option for a ganglion inside bone and present very close to the suprascapular nerve.
